# Unlipidated Outer Membrane Protein Omp16 (U-Omp16) from *Brucella* spp. as Nasal Adjuvant Induces a Th1 Immune Response and Modulates the Th2 Allergic Response to Cow’s Milk Proteins

**DOI:** 10.1371/journal.pone.0069438

**Published:** 2013-07-05

**Authors:** Andrés E. Ibañez, Paola Smaldini, Lorena M. Coria, María V. Delpino, Lucila G. G. Pacífico, Sergio C. Oliveira, Gabriela S. Risso, Karina A. Pasquevich, Carlos Alberto Fossati, Guillermo H. Giambartolomei, Guillermo H. Docena, Juliana Cassataro

**Affiliations:** 1 Laboratorio de Inmunogenética, INIGEM-CONICET, Hospital de Clínicas “José de San Martín”, Facultad de Medicina, Universidad de Buenos Aires (UBA), Buenos Aires, Argentina; 2 Laboratorio de Investigaciones del Sistema Inmune (LISIN), Facultad de Ciencias Exactas, Universidad Nacional de la Plata, Buenos Aires, Argentina; 3 Department of Biochemistry and Immunology, Institute of Biological Sciences, Federal University of Minas Gerais, Belo Horizonte, Minas Gerais, Brazil; 4 Instituto de Estudios de la Inmunidad Humoral (IDEHU-CONICET), Facultad de Farmacia y Bioquímica, Universidad de Buenos Aires (UBA), Buenos Aires, Argentina; Instituto Butantan, Brazil

## Abstract

The discovery of novel mucosal adjuvants will help to develop new formulations to control infectious and allergic diseases. In this work we demonstrate that U-Omp16 from *Brucella* spp. delivered by the nasal route (i.n.) induced an inflammatory immune response in bronchoalveolar lavage (BAL) and lung tissues. Nasal co-administration of U-Omp16 with the model antigen (Ag) ovalbumin (OVA) increased the amount of Ag in lung tissues and induced OVA-specific systemic IgG and T helper (Th) 1 immune responses. The usefulness of U-Omp16 was also assessed in a mouse model of food allergy. U-Omp16 i.n. administration during sensitization ameliorated the hypersensitivity responses of sensitized mice upon oral exposure to Cow’s Milk Protein (CMP), decreased clinical signs, reduced anti-CMP IgE serum antibodies and modulated the Th2 response in favor of Th1 immunity. Thus, U-Omp16 could be used as a broad Th1 mucosal adjuvant for different Ag formulations.

## Introduction

The main function of the mucosa is to maintain normal physiology while discriminating between dangerous and innocuous proteins or organisms [1]. Thus the induction of mucosal immune responses is of paramount importance in both health and disease.

Vaccination through the mucosal route is an interesting strategy for antigen (Ag) administration because it is not associated with pain or stress, and its administration is very easy and cost-efficient. Induction of immune responses following mucosal immunization -using non-live vaccines-is usually dependent upon the co-administration of appropriate adjuvants that can initiate and support the transition from innate to adaptive immunity [2].

An adjuvant is a vaccine component that, through its capacity to act as an immunomodulator/immunostimulant induces and/or enhances an immune response against co-delivered Ags. While there are many types of adjuvants, not all of them are effective at promoting mucosal immune responses. In fact, alum, the most common adjuvant used in current human vaccines, is a poor inducer of mucosal immunity. Possibly the most studied mucosal adjuvants are the bacterial derived ADP-ribosylating enterotoxins, including cholera toxin (CT), heat-labile enterotoxin from *Escherichia coli* (LT), and their mutants or subunits [3]. These enterotoxins promote the induction of antigen-specific IgA antibodies and long-term memory against co-administered antigens when delivered by mucosal or transcutaneous route [2]. However, safety issues have prevented full realization of the potential of this type of mucosal adjuvants. Intranasal (i.n.) immunization, even with low-toxicity mutants, can induce Bell’s palsy [4] and oral administration with these toxin mutants induce poor immunogenicity, as with the B-subunit alone. Therefore, at present much work is being directed towards the development of new low toxicity toxin derivates.

Another type of mucosal adjuvants are Toll-like receptor (TLR) agonists [5]. These ligands activate these pathogen recognition receptors, promoting intracellular signaling, cytokine release and immune cell activation. Recently, monophosphoryl lipid A was the first TLR agonist used in a human vaccine formulation: the FDA approved human papillomavirus vaccine, Cervarix^TM^, by GlaxoSmithKline [6–8].

As the complex nature of mucosal immune induction is understood promising new mucosal adjuvants can be discovered [1]. A high-quality adjuvant would be of relevance not only in vaccines against infectious diseases but also for the control of allergic diseases. Currently, allergic diseases represent a major health problem in industrialized countries. A common feature of these diseases is the production of allergen-specific IgE against normally innocuous food and environmental Ags. Therefore, the majority of new interventions try to control the overexpression of Th2 cytokines or skew the Th1: Th2 balance towards a Th1 profile [9,10]. Unfortunately, although many treatments for allergic diseases and anti-IgE antibody therapies exist, these require a long term recurrent administration of drugs [11].

Milk allergy is one of the most common food allergies with a prevalence of 2.5% among children and 0.3% in adults [12]. There are different classifications of milk allergies: IgE-mediated and non-IgE-mediated disorders [13]. Non-IgE-mediated milk allergy is generally not considered life-threatening, while IgE-mediated milk allergy has been implicated in anaphylactic episodes, being milk the third most common food responsible for severe food-induced anaphylactic reactions in young children (8%-15% cases) [14,15]. The IgE-mediated milk allergy involves production of IgE antibodies upon first exposure to milk protein leading to sensitization of mast cells. Subsequent exposures to the same milk Ags result in a crosslinking of mast cells bound-IgE, leading to activation and release of inflammatory mediators.

Previously, we reported that unlipidated outer membrane protein of 16 kDa from *B. abortus* (U-Omp16) is a new *Brucella* pathogen associated molecular pattern (PAMP) that activates dendritic cell (DCs) *in vivo* and has self-adjuvanting properties when administered by the oral or intraperitoneal route [16]. Taking into account these previous results, we hypothesized that U-Omp16 would be a useful adjuvant in mucosal vaccine formulations. In this work we studied the mucosal adjuvant capacity of the protein U-Omp16 when is co-administered with a model Ag (OVA) by the nasal route and also assessed its capacity to modulate milk allergy in mice.

## Results

### U-Omp16 induces inflammatory cell recruitment to bronchoalveolar lavage (BAL) and Ag internalization

**Figure 1:**
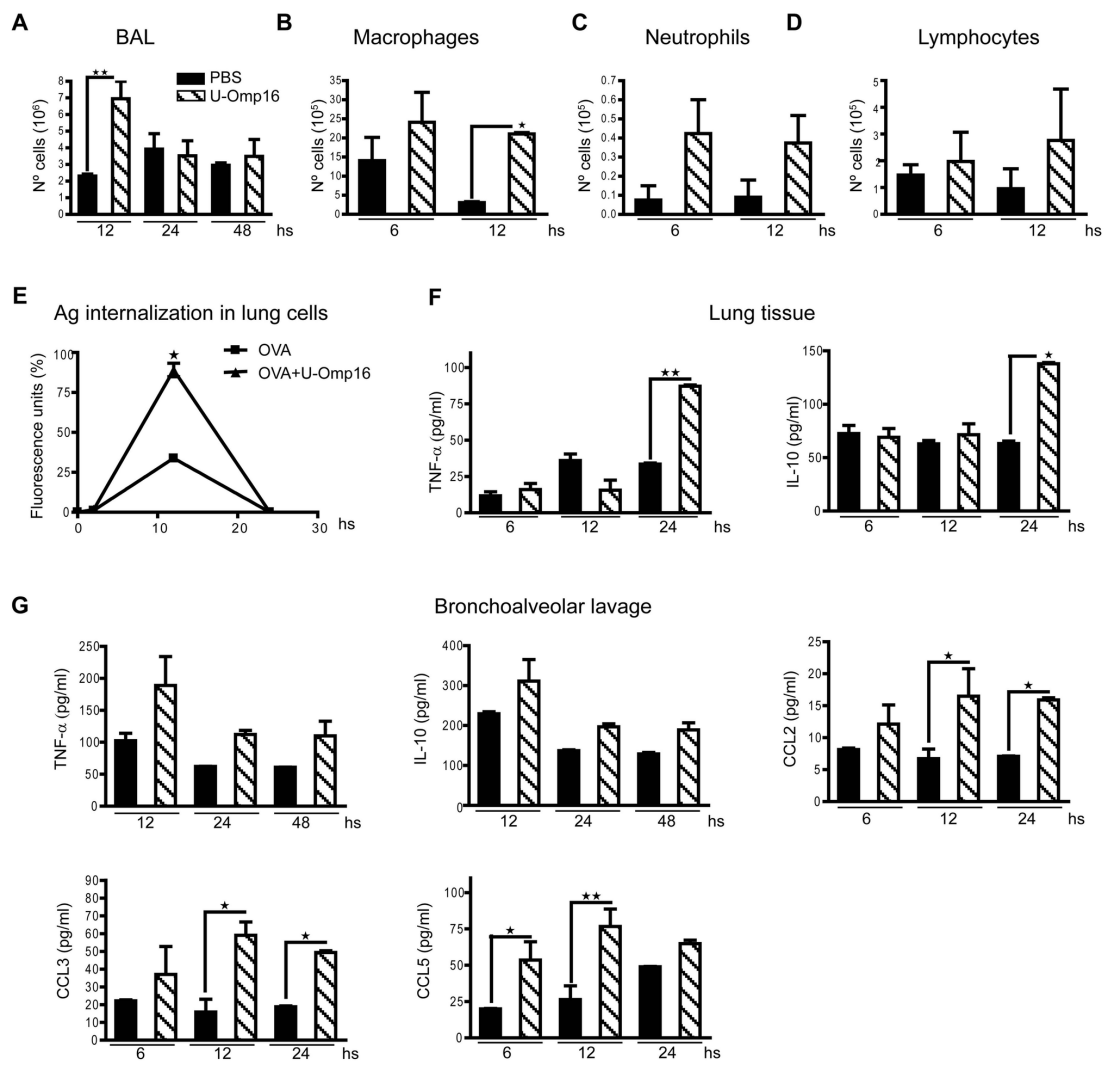
Inflammatory response induced after nasal delivery of U-Omp16. C57BL/6 mice (*n*=5/group) were intranasally administered with U-Omp16 or PBS as control and the number of total cells (**A**) and differential counts of macrophages (**B**), neutrophils (**C**) and lymphocytes (**D**) were determined in BAL at different time points. (**E**) Animals were intranasally administered with OVA_(AF647)_ alone or OVA_(AF647)_ plus U-Omp16. Lungs were obtained at different time points after delivery and the emission of fluorescence was evaluated in cell suspensions from each lung (1 x10^6^ cells). (**F**) C57BL/6 animals were administered through the nasal route with U-Omp16 or PBS as control and at different times post administration the level of TNF-α, IL-10 in lung tissues, and (**G**) TNF-α, IL-10, CCL2, CCL3 and CCL5 in BAL were determined by ELISA. Samples were assayed in duplicated and data represent the mean ±SEM from each group of five mice, (**P<0.001, *P<0.05 *vs* PBS group). These results are representative of 3 independent experiments with similar results.

Inflammatory cells initiate and drive adaptive immune responses. To determine if U-Omp16 possesses the capacity to recruit immune cells, mice were administered through the i.n. route with U-Omp16 or PBS alone as control. BAL was obtained at 12, 24 and 48 h following administration and total cells were counted. U-Omp16 induced a significant increase in the total cell number recruited to the BAL at 12 h (6.9x10^6^ cells, *P*<0.01 *vs* PBS group) (Figure 1A). However at 24 and 48 h post administration there were no significant differences in the number of total cells between U-Omp16 and PBS immunized groups. We then studied the recruitment of macrophages, neutrophils and lymphocytes to BAL after nasal delivery of U-Omp16 or PBS as control. As shown in Figure **1B**, nasal administration of U-Omp16 induced the recruitment of macrophages at 12 h post delivery in comparison with PBS administered group. A slight but non-statistically significant increase in neutrophils at 6 and 12 h and in lymphocyte number at 12 h post delivery was observed in BAL from U-Omp16 administered mice (Figure 1C and D).

Some adjuvants function by increasing the Ag’s half-life or directing and increasing Ag internalization. This prompted us to examine if U-Omp16 was able to increase the delivery of the Ag in lungs after i.n. administration. Ag fate was studied using ovoalbumin (OVA) conjugated with a fluorescent dye –Alexa Fluor 647-, thus fluorescence intensity is proportional to the amount of internalized Ag. To this end, animals were i.n. administered with PBS, OVA_(AF647)_ alone or OVA_(AF647)_ plus U-Omp16. Two, 12 and 24 hours after administration, lung tissue cells were obtained from every animal and fluorescence intensity was determined in a fluorescence plate reader. We observed a rise in fluorescence intensity in lung cells derived from animals immunized with OVA_(AF647)_+U-Omp16 in comparison with mice immunized with OVA_(AF647)_ alone (Figure 1E). Altogether these results indicate that U-Omp16 induces the recruitment of inflammatory cells 12 h after nasal administration and increases the amount of Ag inside lung cells.

### U-Omp16 induces the production of cytokines and chemokines in BAL and lungs

To study the innate immune response induced by nasal delivery of U-Omp16, we measured the level of pro- and anti-inflammatory cytokines and chemokines involved in the recruitment of different cell subtypes (monocytes, neutrophils and lymphocytes). To this end, animals were i.n. administered with U-Omp16 or PBS as negative control and at different time points after administration mice were euthanized. Then TNF-α, IL-10, CCL2, CCL3 and CCL5 production were determined in BAL by ELISA. TNF-α and IL-10 production were also measured in lung tissues. As observed in Figure **1**, nasal administration of U-Omp16 induced a significant increase in the production of TNF-α and IL-10 (*P*<0.001 and *P*<0.05 *vs* PBS group) in lung tissues 24 h post delivery (Figure 1F). Besides, a slight but non-statistically significant increase in the production of TNF-α and IL-10 was observed at 12 h post U-Omp16 i.n delivery in BAL. In agreement with the non-significant recruitment of neutrophils the amount of KC and myeloperoxidase did not significantly increase in BAL from U-Omp16 administered mice (data not shown). Besides, U-Omp16 induced the production of pro-inflammatory chemokines CCL2, CCL3 at 12 and 24 h post delivery in BAL while CCL5 at 6 and 12 h (*P*<0.05 *vs* PBS group) (Figure 1G). These results indicate that U-Omp16 stimulates the production of pro-inflammatory cytokines (TNF-α) and chemokines (CCL2, CCL3 and CCL5) and anti-inflammatory cytokines (IL-10) at the lung.

### U-Omp16 co-administered with OVA through the nasal route induces a Th1 immune response

Next we investigated if nasal co-delivery of U-Omp16 with the model Ag OVA would promote adaptive immune responses. For this, mice were i.n. immunized with OVA plus i) PBS, ii) U-Omp16 or iii) U-Omp16 completely digested with proteinase K. Three weeks post vaccination animals were sacrificed, spleen cells were obtained and cultured with OVA or complete medium.

**Figure 2:**
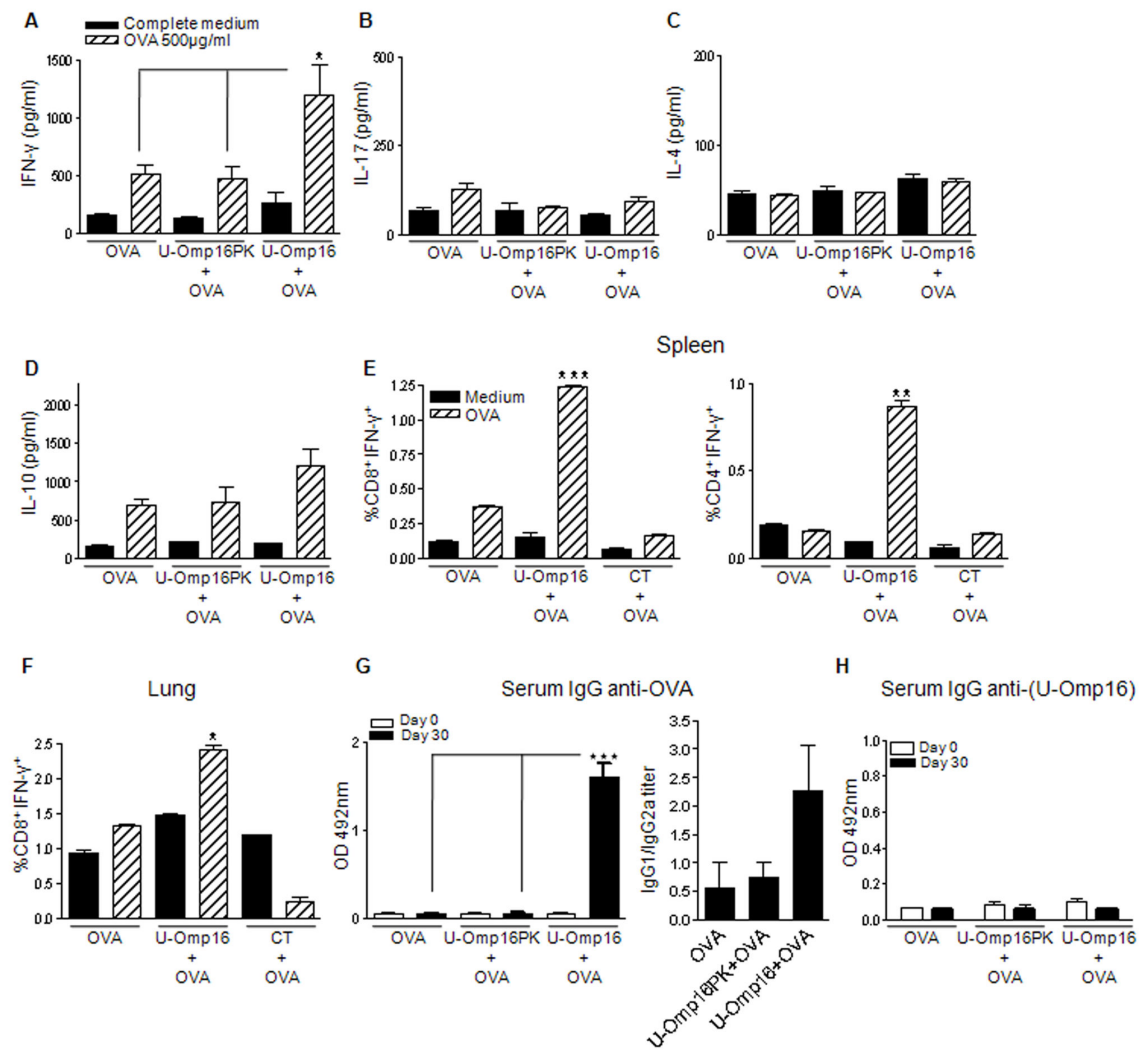
U-Omp16 induces a T helper 1 immune response when administered as nasal adjuvant. C57BL/6 mice were immunized by the nasal route with: OVA plus (i) PBS or ii) U-Omp16 previously digested with proteinase K (U-Omp16PK) or plus (iii) U-Omp16. Three weeks after last immunization animals were sacrificed and spleen cells were stimulated *in vitro* with OVA 500 µg/ml or complete medium (RPMI). Culture supernatants were harvested 5 days after stimulation and cytokine concentration of (**A**) IFN-γ, (**B**) IL-17, (**C**) IL-4 and (**D**) IL-10 (pg/ml) were determined by ELISA. U-Omp16 when administered as nasal adjuvant stimulates the induction of CD4^+^ and CD8^+^ OVA-specific T cells that produce IFN-γ. Percentages are represented for spleen (**E**) CD8^+^ or CD4^+^ T lymphocytes, and lung CD8^+^ T cells (**F**) expressing IFN-γ. (**G**) Anti-OVA IgG was determined in sera from immunized animals on days 0 (pre-immune) and 30 (post-immune) by indirect ELISA. Data represent the mean ±SEM from each group of five mice; (***P<0.001, **P<0.01, *P<0.05 *vs* OVA group). These results are representative of 3 independent experiments with similar results.

Upon stimulation with OVA, splenocytes from U-Omp16+OVA-immunized mice showed a significant production of IFN-γ (*P*<0.05 *vs* OVA or U-Omp16+PK groups). Splenocytes from animals immunized with U-Omp16 previously digested with proteinase K plus OVA induced similar levels of IFN-γ production upon Ag stimulation to OVA immunized group (Figure 2A). These results indicate that U-Omp16’s adjuvant capacity resides in the protein moiety and is not due to any other non-protein contaminant present in the U-Omp16´s preparation. There were no significant differences in IL-17, IL-4 nor IL-10 secretion upon Ag stimulation between all the immunized groups (Figure 2B–D). Taken together, these results indicate that U-Omp16 used as a nasal adjuvant drives the immune response towards a Th1 profile.

To expand upon these findings we decided to investigate which cells are the sources of IFN-γ upon Ag stimulation. Hence, splenocytes and lung cells from immunized animals were obtained and stimulated or not with OVA. Nasal co-delivery of U-Omp16 as adjuvant induced an increase in the frequency of CD8^+^- and CD4^+^- IFN-γ-producing T cells (1.23% and 0.89%, respectively) in spleens (Figure 2E) while an increase in IFN-γ producing CD8^+^ T cells (2.32%) at lungs (Figure 2F) upon OVA stimulation. When CT has been used as nasal adjuvant with OVA, there was no increase in the frequency of spleens or lungs IFN-γ producing T cells upon Ag-stimulation (Figure 2E and F). Additionally, the humoral immune response was evaluated measuring OVA-specific IgG levels in serum from immunized mice. Co-administration of U-Omp16 with OVA increased OVA-specific IgG in comparison to animals vaccinated with OVA alone or with OVA plus U-Omp16+PK (Figure 2G). U-Omp16 as nasal adjuvant induced anti-OVA IgG1 and IgG2a serum antibodies with a slight bias on IgG1 versus IgG2a secretion (IgG1/IgG2a ratio=2) (Figure 2G). Of note, U-Omp16 nasal co-delivery with OVA did not induce serum IgG responses against U-Omp16 (the adjuvant) (Figure 2H).

Overall, these results demonstrate that the administration of U-Omp16 as nasal adjuvant with OVA, as model Ag, induces Ag-specific Th1 immune responses with systemic Ag-specific IgG production. IFN-γ production is mediated by CD4^+^ and CD8^+^ T cells systemically (spleens), and by CD8^+^ T cells at the mucosal site (lungs).

### U-Omp16 does not induce a pathological immune response at the lung or CNS after administration or vaccination

To evaluate the safety profile of U-Omp16 at the lung and CNS different experiments were conducted. Histological sections of lungs and brains from immunized mice were obtained, stained with H&E and evaluated by a pathologist. In coincidence with the recruitment results showed in Figure 1, a transient infiltration of immune cells in perivascular region at 12 h post U-Omp16 (Figure **S1A**) or U-Omp16+OVA (Figure **S1C**) nasal delivery was observed at the lung, while tissue morphology was not affected with any pathological changes induced. At 24 h post U-Omp16+OVA nasal delivery the infiltration diminished remaining similar to OVA-delivered group (Figure S1D). At 2 weeks post nasal U-Omp16 delivery no cellular infiltrate was observed and the lung showed complete resolution of the inflammation with no apparent infiltration or edema as in the control with PBS (Figure **S1B**). Lung histology was also studied after a second i.n administration of U-Omp16 (2 weeks after 2 doses of U-Omp16+OVA) and there was no alteration of the lung tissue architecture, thus indicating that no pathological adverse reaction is promoted even in the presence of a memory immune response (Figure **S1E**). In contrast, CT co-administration induced a prominent infiltration with vessel congestion, edema, microhaemorrhage foci and thickened septums (Figure **S1C-D**). Therefore, we conclude that U-Omp16 produce no lung remodeling with a transient inflammation followed by a spontaneous recovery of the tissue architecture. Moreover, no inflammatory reaction was observed at the olfactory bulb neither after U-Omp16 nasal administration (12 h and 2 weeks after) nor after U-Omp16+OVA nasal immunization (2 weeks after 2 doses) (Figure **S2E**).

We also studied if OVA or U-Omp16 can reach CNS (olfactory bulb, forebrain and posterior brain) after nasal administration of OVA+U-Omp16 at different time points after delivery (2, 12 and 24 h). Different experimental methods were used to assess the presence of antigen and adjuvant in CNS tissues after the nasal delivery: i) by Western Blot with an anti-OVA antibody or with U-Omp16-specific antiserum and ii) fluorescence quantification (using FITC-labeled OVA). Neither OVA nor U-Omp16 in the different samples analyzed was detected (Figure **S2A–D**) indicating that after nasal delivery no vaccine component reached the CNS.

### U-Omp16 reduces the symptoms in a food allergy mouse model

**Figure 3:**
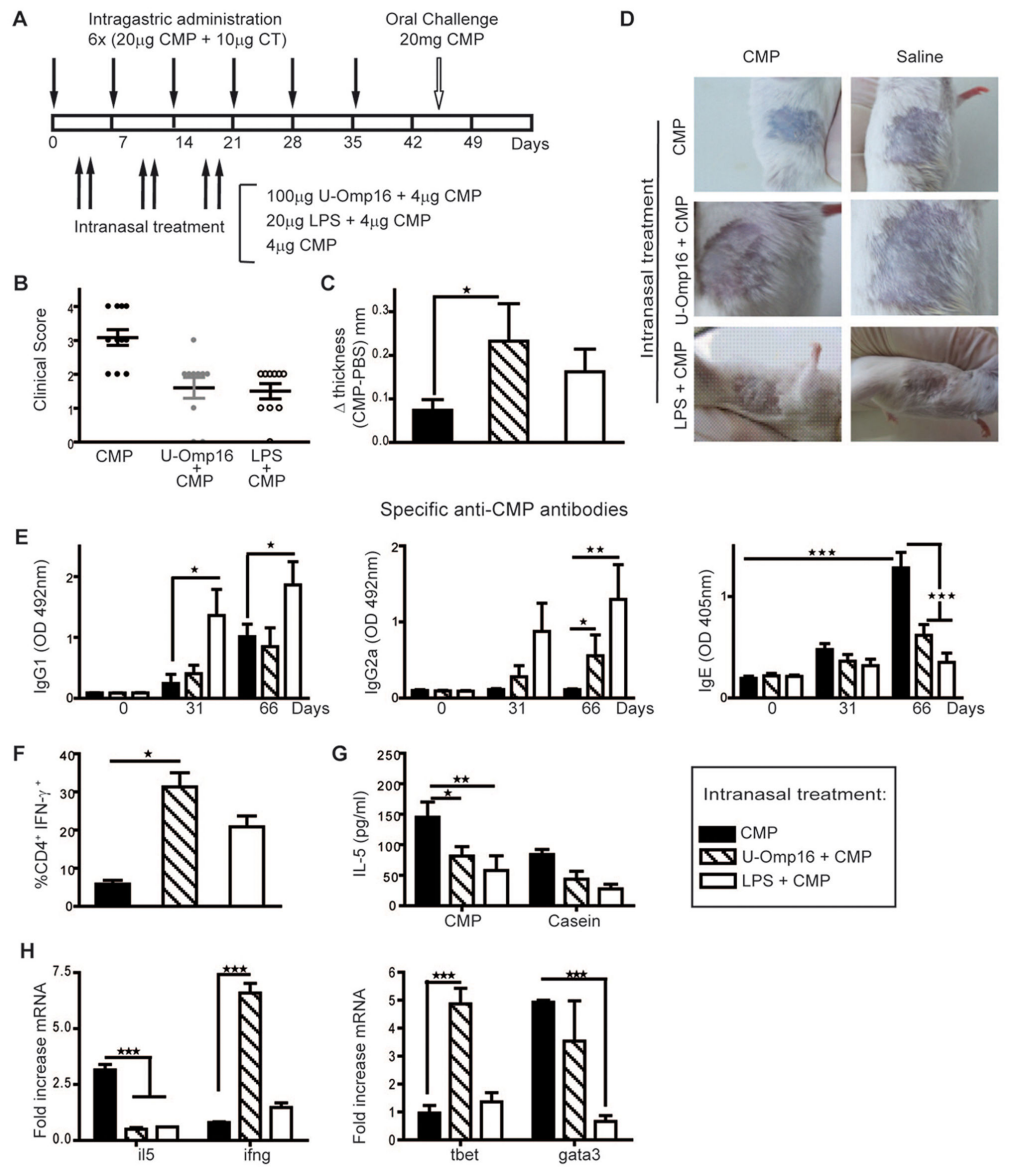
U-Omp16 administrated *in* *vivo* modulates the allergic reaction in a mouse model of food allergy. (**A**) Outline of the experimental design for the mouse model of food allergy in BALB/c mice (*n*=10/group). (**B**) Hypersensitivity scores of sensitized and sensitized/treated mice 30 minutes following the i.g. challenge with CMP (*n/group=10*). (**C**) Delayed-type hypersensitivity (DTH) response to CMP was assayed 3 weeks after the last boost to evaluate the cellular immune response *in vivo*. Twenty µg of CMP were injected into one footpad, and saline was injected into the contra lateral footpad, as a negative control. The thickness of both footpads was measured 48 h later. (**D**) Cutaneous test in sensitized and treated mice to evaluate the induction of immediate inflammation. (**E**) Determination of CMP-specific serum IgG1, IgG2a and IgE after oral CMP sensitization and intranasal treatment. (**H**) Treatment with U-Omp16 stimulates the induction of CD4^+^ CMP-specific T cells that produce IFN-γ. Spleen cells from CMP sensitized and treated mice were stained with specific anti-CD4 (PE) monoclonal Ab. After permeabilization cells were stained with anti-IFN-γ (FITC) or isotype control (FITC) monoclonal Abs for intracellular flow cytometry analysis. (**G**) Splenocytes were collected 24 h after the oral challenge and stimulated *in vitro* with CMP (350 µg/ml) or casein (200 µg/ml) for 72 h. Levels of IL-5 in culture supernatants of spleen cells from sensitized and treated mice were determined by ELISA. (**I**) mRNA expression for cytokines (IL-5 and IFN-γ) and transcription factors (t-bet and gata-3) was quantified 24 h after oral challenge in jejunum segments. Data are expressed as mean values ±SEM (***P<0.001, **P<0.01, *P<0.05 *vs* CMP treated group). These results are representative of two independent experiments with similar results.

To confront our results obtained with OVA with a relevant food allergen and to study its potential application in allergy vaccines, we evaluated the U-Omp16’s adjuvant capacity to prevent the induction of a specific allergic reaction in a mouse model of food allergy. To conduct these experiments, mice were sensitized by gavage with Cow’s Milk Protein (CMP) +CT while simultaneously administered i.n with CMP alone or with LPS plus CMP as treatment control, or with U-Omp16 plus CMP. In Figure **3A** a schematic representation of the sensitization schedule is depicted. The clinical signs recorded following the oral challenge were scored and are shown in Figure **3B**. Animals treated i.n. during sensitization with CMP alone showed a higher score level as compared to treated mice with CMP and adjuvants. The use of LPS or U-Omp16 with the co-administered Ag rendered lower scores (average score 1.5 for Treat LPS, 1.55 for Treat OMP 16 and 3.1 for Treat CMP) indicating that the use of U-Omp16 ameliorates the hypersensitivity responses of sensitized mice upon oral exposure to Ag.

We next addressed the ability of the different treatments to prime a Delayed-type hypersensitivity (DTH) response, as a reflection of the CD4^+^ T cell ability to induce a Th1-mediated immunomodulation. As shown in Figure **3C** a higher DTH response was observed in mice that received U-Omp16 or LPS as adjuvant, as compared to that in sensitized mice that received CMP alone as treatment. Saline injected in the contralateral hint footpad rendered a negligible swelling. The average scores for each group were U-Omp16= 0.21mm, LPS= 0.17mm and CMP= 0.08mm.

To investigate if this suppressed reaction could be linked to a milder allergy the presence of IgE bound to skin mast cells was assessed by cutaneous tests. Figure **3D** shows the results of the skin challenge with CMP, and as it can be seen, an immediate extravasation of the blue dye was only achieved in sensitized mice treated with CMP in vehicle. No increase in vascular permeability was observed in mice treated with U-Omp16 or LPS.

Overall, these results indicate that U-Omp16 modulates the CMP-specific allergic immune response *in vivo*, preventing the clinical reaction against oral exposure to the Ag.

### Intranasal immunization with U-Omp16 promotes a decrease in the IgE antibody response and an increase in the IgG2a level

To investigate if U-Omp16 promotes a specific Th1-mediated immune response that modulates the allergic state we evaluated the immunoglobulin isotypes by immunoassays. Figure **3E** shows the kinetics of induction of CMP-specific IgG1, IgG2a and IgE immunoglobulins. During the sensitization phase CMP-specific IgE and IgG1 were induced, which reflects that a Th2-immune response is triggered against CMP with the use of CT through the oral route. In contrast, when U-Omp16 plus CMP was administered, specific CMP IgG2a antibodies were induced, whereas IgG1 remained unchanged, and remarkably, IgE was down-modulated. As expected, LPS administration induced an increase in both IgG1 and IgG2a in comparison with the control treatment group. The IgG1/IgG2a ratio, which indicates whether a Th1 or a Th2 type immune response prevails, suggested that U-Omp16 induced *in vivo* a bias towards a Th1 immune profile.

### U-Omp16 promotes spleen CD4^+^ T cells to produce IFN-γ upon CMP stimulation and imprints a mucosal Th1 immune profile

To confirm that U-Omp16 treatment was able to induce a CMP-specific Th1 response we investigated the cytokine production of splenocytes and mRNA expression in jejunum from mice treated through the nasal route. Co-administration of CMP with U-Omp16 or LPS induced an increase in the frequency of CMP-specific IFN-γ producing CD4^+^ T cells while reduced IL-5 secretion by spleen cells compared to CMP treated-mice (Figure 3F and G), indicating the Th1-shifted immune response.

To further characterize the response induced in the gut mucosa after U-Omp16 nasal treatment, gata-3 and t-bet gene expression in jejunum of treated mice were determined. As shown in **Figure 3H** U-Omp16 modulated the *ifn-γ* and *il-5* gene expression in jejunum of mice treated with this adjuvant, as compared to mice that received only CMP as treatment. Furthermore, a local increased *t-bet* gene expression that fitted with the Th1-biased immune response induced was evidenced. In contrast, *gata-3* expression remained similar to CMP treated animals.

Cholera Toxin plus CMP administration induces a mild inflammation in the gut mucosa with a mononuclear infiltrate. There were no significant differences in the cellular composition of the gut mucosa between the groups (data not shown).

Overall these results provide strong evidence indicating that U-Omp16 administered at a distant mucosal site is a Th1 immune inducer that can modulate the Th2-mediated allergic sensitization.

## Discussion

Given their potent immunostimulatory capacity, bacterial-derived substances constitute a major potential source of adjuvants. In a previous work we discovered that U-Omp16 from *Brucella* is a new PAMP that signals through TLR4 and has self-adjuvanting properties when delivered either parenterally or orally [16].

In this work we demonstrate that nasal co-delivery of Ag with U-Omp16 induces Ag-specific Th1 responses at systemic (spleen) and mucosal (lungs and gut) levels. Also, U-Omp16 improved systemic humoral immune. The IFN-γ production induced with OVA administration is mediated by CD4^+^ as well as CD8^+^ T cells. The adjuvant capacity could not be due to LPS contamination, since U-Omp16 preparations were exhaustedly depleted of LPS with polymyxin B sepharose, as assessed by LAL assay. Moreover, U-Omp16 lost its Th1 adjuvant capacity when it was completely digested with proteinase K, indicating that the *in vivo* properties of U-Omp16 are in fact due to this protein rather than to another non-protein contaminant. In addition, there were no OVA-specific Th2 responses after U-Omp16 nasal delivery. It has been reported that i.n. immunization leads *per se* to Th17-biased immune responses, regardless of the adjuvant used [17]. However, in this work, we did not find *in vitro* IL-17 release from stimulated splenocytes derived from mice i.n. administered with OVA plus U-Omp16. Based on our present results, it seems that the vaccine formulation, rather than the route of delivery, drives the T helper profile.

The induction of Th1 and CD8^+^ T cell responses is highly desirable, for example, in vaccines targeting either chronic viral diseases, infections linked to intracellular pathogens, cancer (therapeutic vaccines) [18] or to modulate allergy diseases [11,19]. Thus, U-Omp16 would be a suitable adjuvant to be used in these conditions. Besides, U-Omp16 induces a Th1 immune response similar to the one elicited after CpG co-administration (Figure **S3**). Although the TLR9 agonist CpG seemed quite promising, being a strong Th1 adjuvant, clinical studies showed that vaccines containing CpG cause a severe autoimmune disease, Wegener’s granulomatosis [20]. In addition toxin based adjuvants such us CT or CTB are redirected to the olfactory bulb in the CNS when administered via the nasal route, which has raised concern about adverse effects at the CNS. For this reason, the search for new mucosal adjuvants that preferentially induce Th1 responses is worth the effort. In this work we demonstrated that U-Omp16 nasal delivery does not induce an inflammatory response at the CNS and most important neither the Ag (OVA) nor this adjuvant (U-Omp16) reached the CNS after nasal delivery.

Adjuvants are thought to exhibit different modes of action [21,22]. For example, certain adjuvants are able to convey long-term presentation of the Ag (depot effect); others induce an inflammatory response by recruitment of immune cells and by helping to target them (e.g. by delivering Ags to APCs). Other adjuvants are capable of enhancing the levels of co-stimulatory molecules on APCs [23,24]. U-Omp16 has been shown to activate DCs *in vitro* and *in vivo* [16]. In this work we found that U-Omp16 delivered by the nasal route induces the production of chemokines and the recruitment of inflammatory cells at BAL and lungs, which are responsible for the induction of an inflammatory environment. In this way, i.n administration of U-Omp16 drives the production of TNF-α and IL-10 in lung tissues cells 24 h post delivery. Possibly, this early TNF-α production is involved in the induction of the required inflammatory context for the initiation of adaptive mucosal immune responses. Adjuvants should not elicit unacceptable local reactions, when used in prophylactic and therapeutic vaccines [25]. IL-10 production -which is involved in anti-inflammatory processes-, would be an important feature of this adjuvant because excessive inflammatory responses would be undesirable. U-Omp16 i.n administration induced an increase in the number of DCs (CD11c^+^CD11b^-^ and CD11c^+^CD11b^+^) and monocytes/macrophages (CD11b^+^CD11c^-^) in lungs from 2 to 18 h post-inoculation (Figure **S4A**). As IL-10 and TNF-α are produced after U-Omp16 stimulation of DCs (BMDCs) and macrophages (BMDMs) *in vitro* (Figure **S4B**), we speculate that DCs and monocytes are the source of IL-10 and TNF-α *in vivo*. Also, U-Omp16 can be internalized by DCs (BMDCs) *in vitro* (Figure **S4C**). In coincidence, i.n delivery of U-Omp16 is able to induce an increase in the amount of co-delivered Ag inside lung cells. This ability may result on a reduction of Ag dose and may help to reduce vaccine costs.

Worth mentioning, histological studies of lungs were conducted. In coincidence with the recruitment studies a transient recruitment of immune cells post U-Omp16 nasal delivery was observed. Remarkably the tissue histology was not affected. At later time points following administration or nasal vaccination no cellular infiltrate was observed and the lung architecture was normal, indicating that no pathological adverse reaction was induced by U-Omp16, even in the presence of a memory immune response.

Based on the above-mentioned results, we decided to test U-Omp16’s adjuvant capability to modulate milk allergy in mice. Cow’s milk allergy is a global health concern that occurs more frequently among children than adults. At present, there are no definitive therapeutic options for food allergy patients. Once a food allergy is diagnosed, the standard of care includes strict elimination of the allergen from the diet and ready access to injectable epinephrine [12,26]. Thus, the induction of allergen-specific Th1 responses has been proposed as a promising concept for treatment of Th2-biased hyper-responsiveness. The intranasal co-administration of U-Omp16 plus CMP during sensitization decreased clinical signs of hypersensitivity after oral challenge with the allergen in sensitized mice. In addition, clinical score values in U-Omp16 administered mice are similar to LPS-treated group, known as a strong mucosal modulator of this response. Furthermore, a significant DTH response is induced in animals i.n. delivered with U-Omp16 or LPS, indicating that the administration of U-Omp16 during sensitization as an adjuvant can bypass the mucosal tolerance mechanisms. Moreover, U-Omp16 administration during sensitization induces a reduction in allergen-specific serum IgE and after the cutaneous test no increase in vascular permeability was observed. These results enabled us to assume that animals treated with adjuvants during sensitization have skin mast cells with no detectable specific IgE antibodies attached to the cell membrane. This situation might be extended to other mucosal mast cells, and reflects that further exposure to Ag promotes no hypersensitivity reaction.

To enforce these findings, we found that i.n. delivery of U-Omp16+CMP during sensitization induces an increase in the frequency of spleen CD4^+^ T cells that produce IFN-γ upon *in vitro* CMP stimulation. Moreover, U-Omp16 increases *ifn-γ* and *t-bet* gene expression at jejunum that correlates with the Th1 biased immune response induced. These results account for the ameliorated clinical signs observed after oral allergen challenge with CMP in animals treated with U-Omp16+CMP during sensitization. Finally, these findings confirm that the nasal delivery of U-Omp16 modulates the specific immune response at the mucosa and systemically.

Of note, U-Omp16 Th1 mucosal adjuvant capabilities have been demonstrated using C57BL6 mice (OVA model) and BALB/c mice (CMP model), suggesting that U-Omp16´s activity is independent on the genetic background, therefore strengthening and expanding its potential as mucosal adjuvant.

Overall, this work shows that U-Omp16 is a Th1 immune response inducer that can modulate the Th2 mediated allergic sensitization and supports U-Omp16’s potential as a broad Th1 mucosal adjuvant for different Ag formulations in line with the present requirements.

## Materials and Methods

### Ethics Statement

All experimental protocols of this study were conducted in strict agreement with international ethical standards for animal experimentation (Helsinki Declaration and its amendments, Amsterdam Protocol of welfare and animal protection and National Institutes of Health, USA NIH, guidelines: Guide for the Care and Use of Laboratory Animals). All surgeries were conducted under sodium pentobarbital anesthesia. The protocols of this study were approved by the Institutional Committee for the Care and Use of Laboratory Animals from Institute of Immunology, Genetics and Metabolism

INIGEM-CONICET, University of Buenos Aires (Permit Number: 102).

### Mice

Female eight week old C57BL/6 and BALB/c mice were purchased from the School of Animal Science at the National University of La Plata (La Plata, Argentina). Mice were housed in appropriate conventional animal care facilities and handled according to international guidelines required for animal experiments.

### Antigens and adjuvants

OVA grade V (Sigma Aldrich) was dissolved in sterilized saline solution. The recombinant unlipidated (U-) Omp16 was expressed and purified as previously described [27,28]. Protein concentration was determined by the bicinchronic acid assay (Pierce, Rockford, IL). Lipopolysaccharide (LPS) contamination was adsorbed with Sepharose-polymyxin B (Sigma-Aldrich, St. Louis, MO). Endotoxin determination was performed with Limulus amoebocyte chromogenic assay (LONZA, Argentina). All U-Omp16 preparations contained less than 0.10 endotoxin U/mg protein.

In some experiments U-Omp16 was enzymatically digested to be used as control. For this, U-Omp16 was treated with proteinase K-agarose from *Tricirachiumalbum* (Sigma-Aldrich) for 2 h at 37°C, following the manufacturer’s indications. The enzyme immobilized in agarose was then centrifuged out (2000 x g, 5 min), and the supernatants were incubated for 1 h at 60°C to inactivate any fraction of soluble enzyme. The complete digestion of the proteins was checked by SDS-PAGE, followed by Coomassie blue staining as was described [16].

### Preparation of single-cell suspensions

Spleens were aseptically removed and single cell suspensions were prepared by gently teasing through a sterile stainless steel screen. To obtain cells from lungs, organs were removed and digested with 400 U/mL Collagenase Type IV and 50 mg/mL DNase in RPMI 1640 for 30 minutes at 37°C. Cell suspensions were filtered through a stainless-steel sieve, and were washed twice in PBS solution.

### Bronchoalveolar lavage (BAL)

BAL from mice was obtained as described previously [29] centrifuged and supernatants were immediately used to detect cytokines and chemokines. Cell pellets were suspended in PBS containing 3% bovine serum albumin. Total leucocytes were counted with a hemocytometer, and the percentages of different leucocytes were determined using standard morphological criteria, examining cytospin slides by May-Grund-Wald and Giemsa staining.

### Determination of cytokine and chemokine production in BAL and lung tissues after U-Omp16 nasal delivery

C57BL/6 mice were i.n. administered with PBS or U-Omp16 (20 µg) and 6, 12, 24 and 48 h after delivery BAL was obtained from every mouse. The amount of cytokines and chemokines was evaluated as previously described [29]. The concentration of TNF-α, IL-10, CCL-2, CCL-3 and CCL-5 in BAL and lung supernatants was analyzed by ELISA, according to the manufacturer’s instructions (Pharmingen, San Diego, CA).

### Determination of Ag internalization in vivo

OVA Alexa Fluor 647 –OVA _(AF647)_ (Molecular Probes, USA) was used as Ag. Animals were i.n. administered with OVA_(AF647)_ (50 µg), plus i) PBS or ii) U-Omp16 (20 µg). After administration lung cells were obtained at different time points and washed. The emission of fluorescence was measured in cell suspensions (1x10^6^ cells/well) using a fluorescence plate reader (Victor3, PerkinElmer, Waltham, MA).

### Immunization and experimental design

C57BL/6 mice were i.n. immunized once a week during 3 weeks with i) OVA (50 µg), ii) OVA (50 µg) plus U-Omp16 (20 µg) or iii) OVA (50 µg) plus U-Omp16 (20 µg previously digested with proteinase K) in 20 µl per nostril. In some experiments a group of animals was also immunized with OVA (50 µg) plus CpG (10 µg) as a control. Blood was obtained on day 0 and 30 following the first immunization, and three weeks after the last immunization mice were sacrificed to perform cellular *in vitro* experiments.

### Determination of the T helper immune responses

Single spleen cell suspensions from immunized and control mice were cultured in duplicate at 4 x10^6^ cells/ml in RPMI 1640 (Life Technologies BRL, Grand Island, NY) supplemented with 10% FCS (Life Technologies), 1 mM sodium pyruvate, 2 mM L-glutamine, 100 U penicillin/ml, and 100 mg streptomycin/ml (complete medium) or with OVA (500 µg/ml) in complete medium. After 5 days of incubation cell culture supernatants were collected. IFN-γ, IL-4, IL-10 and IL-17 production was evaluated by ELISA kits (Pharmingen, San Diego, CA, R&D Systems, Biocientifica S.A, Argentina).

### Intracellular IFN-γ determination

Lung and spleen cells (4x10^6^ cells/well) were cultured in presence of i) complete medium supplemented with IL-2 (1 U/ml) or ii) OVA (500 µg/ml) plus mitomicin C-treated MO5 cells -B16 melanoma cell line transfected with the ovoalbumin gene- (25:1) plus IL-2 for 18 h. Thereafter, cells were treated with 10 µg/ml brefeldin A and were incubated for 6 h. Surface staining was performed with anti-CD4 (PE-Cy5.5), anti-CD8 (Alexa Fluor 647). Then, cells were permeabilized with saponin buffer and stained with anti IFN-γ (PE) or with the Isotype (PE) control. Data acquisition was performed using BD FACSAriaII and data analysis using FlowJo software.

### Histology and histopathology

Lung and brain inflammation was studied at different times after i.n administration. To assess inflammation at short time lungs and brain were excised at 12 h (or 24 h) after single administration, and at longer times (2 weeks) after administration on days 0 and 7. For this, different formulations were administered. Groups of mice were administered with (i) PBS or (ii) U-Omp16 (20 µg). In other experiments, animals were administered with (i) OVA (50 µg), (ii) OVA (50 µg) + U-Omp16 (20 µg) or (iii) OVA (50 µg) + CT (1 µg). At the indicated time post administration lungs and brains were excised, fixed and preserved in cold sterile *para*-formaldehide 4%. Then fixed lungs and brain were paraffin embedded. Finally, five micrometers thick longitudinal sections of lungs, and frontal sections of brain, were obtained and stained with H&E to assess the degree of inflammation or injury in lungs and brain tissue. The analysis of samples has been made by an expert pathologist of the National Academy of Medicine (Buenos Aires, Argentina).

### Intranasal administration to study Ag fate in CNS

To study if i.n administration of U-Omp16 induces re-direction of Ag to CNS OVAFITC labeled (Invitrogen) was used as model Ag. Animals were i.n administered with (i) PBS, (ii), OVAFITC (50 µg) or (iii) OVAFITC (50 µg) + U-Omp16 (20 µg). At different times post administration (2, 12 and 24 h) animals were sacrificed and brain was excised. Each brain area: olfactory bulb (OB), forebrain (FB) and posterior brain (PB) was obtained and suspensions were prepared. Right hemisphere was used for fluorescence assay. Finally, OVAFITC fluorescence was measured in a fluorometer (Victor3, PerkinElmer, Waltham, MA). Also, OVA or U-Omp16 presence in CNS was assessed by Western blot.

### Western Blot Analysis

The OB, FB and PB derived from the left hemisphere, from different experimental conditions were homogenized using TOTEX lysis buffer. The protein lysates were quantified by Bradford method. After that, 100 µg of protein were separated by SDS/PAGE using 15% acrylamide-bisacrylamide gels and transferred onto polyvinylidene fluoride membranes. These were blocked ON at 4°C with TBS/Tween 0.1%. Then membranes were washed with TBS/Tween 0.05% and incubated with primary antibody anti-OVA (1:2000) and anti-Omp16 (1:4000) ON at 4°C. Membranes were washed with TBS/Tween 0.05%, incubated for 1 h and 30 minutes with secondary antibody horseradishperoxidase-conjugated secondary antibodies (1:2000 and 1:5000 respectively), washed in TBS/Tween 0.05% and then developed with ECL. To confirm equal protein loading of all lanes, the same blots were reprobed with an anti-actin antibody.

### Recruitment of DCs and monocytes

C57BL/6 mice were i.n administered once with (i) OVA (50 µg), (ii) OVA (50 µg) + U-Omp16 (20 µg) or (iii) OVA (50 µg) + CT (1 µg). At different times after administration (2 and 18 h) mice were sacrificed and heart perfused with sterile saline solution. After that, lungs were excised and cell suspensions of the organ were obtained. Cells (6x10^6^) were stained with monoclonal Abs anti-CD11c and CD11b and were analyzed by flow cytometry (FACsAriaII, BD Bioscience).

### BMDCs and BMDMs

DCs and macrophages were generated from bone marrow (BM) mononuclear cells from wild-type C57BL/6 mice. Briefly, femurs and tibiae were collected from mice with 6–12 wk old. After removing bone adjacent muscles, marrow cells were extracted by flushing RPMI 1640 medium through the bone interior. Bone marrow cells were then suspended on DC culture medium (RPMI 1640 medium, 10% heat-inactivated fetal/bovine serum, 1 mM sodium pyruvate, 2 mM L-glutamine, 100 U penicillin/ml, and 100 mg streptomycin/ml, 20 ng/ml GM-CSF) or macrophages culture medium (RPMI 1640 medium, 10% heat-inactivated fetal/bovine serum, 1 mM sodium pyruvate, 2 mM L-glutamine, 100 U penicillin/ml, and 100 mg streptomycin/ml, 20 ng/ml M-CSF), and plated on 6 wells plate (1,5 × 10^6^ cells/2 ml culture medium). On days 3 and 5, the cells were refed. On day 8, cells were harvested and expression of DCs (CD11c^+^, MHC class II (MHCII)^low^ or monocytes (CD11b^+^) markers were analyzed by flow cytometry.

### BMDCs and BMDMs stimulation and U-Omp16 internalization in vitro

To study if DCs and monocyte/macrophages were implicated in the production of pro- and anti-inflammatory cytokines, BMDCs and BMDMs (1x10^6^ cells) were incubated *in vitro* with (i) complete medium or (ii) U-Omp16 (20 or 100 µg/ml) for 20 h. After incubation, supernatants were harvested and concentration of TNF-α and IL-10 was determined by ELISA.

In other experiment internalization of U-Omp16 was assessed. In this assay U-Omp16 FITC labeled (Invitrogen) was used to determine internalization. BMDCs (1x10^6^ cells) were incubated for 15 and 30 minutes *in vitro* with: (i) complete medium, (ii) U-Omp16 (20 µg/ml) or (iii) U-Omp16 (100 µg/ml). After incubation cells were washed and internalization was measured by flow cytometry (FACsAriaII, BD Bioscience).

### Intranasal immunomodulation with U-Omp16 in a Cow´ s Milk Protein food allergy mouse model

BALB/c mice were rendered allergic to CMP as previously described [30]. Briefly, mice received 6 weekly intragastric (i.g.) doses of 20 mg of CMP administered as homogenized commercial non-fat dry milk, plus 10 µg of cholera toxin (CT) (Sigma Aldrich, USA) (sensitized mice). Age-matched naïve mice received 6 weekly i.g. doses of 20 mg CMP without CT (sham control). Mice were fasted for 2 h before sensitization, and 3% sodium bicarbonate solution was given to reduce gastric acidity 30 min before the immunization. Ten days after the final boost mice were i.g. challenged with 20 mg CMP. Blood samples were collected during the sensitization phase and sera were stored at -20 ^°^C until used. To modulate the allergic sensitization, mice received twice a week during 4 weeks through intranasal route 4 µg CMP plus 100 µg U-Omp16 (Treat U-Omp16), 4 µg CMP plus 20 µg of LPS (Treat LPS) as a positive treatment control or 4 µg CMP as negative treatment control (Treat CMP). Twenty-four hours following the oral challenge animals were sacrificed and spleens and jejunum were collected. The experimental design is depicted in Figure **3A** **.**

### In vivo evaluation of the allergic state

#### Assessment of clinical signs

Symptoms were observed between 30 and 60 min after the oral challenge in a blinded fashion by 2 independent investigators. Clinical scores were assigned according to the following range: 0 = no symptoms; 1 = scratching and rubbing around the nose and head; 2 = puffiness around the eyes and mouth, diarrhea, pilar erecti, reduced activity, and/or decreased activity with increase respiratory rate; 3 = wheezing, labored respiration, cyanosis around the mouth and the tail; 4 = no activity after prodding, or tremor and convulsion; and 5 = death.

#### Cutaneous test

Mice were shaved on both flanks and injected intradermically with 200 µg of CMP in 50 µl of sterile saline in one flank, and saline alone in the other flank as negative control. Mice were also injected intravenously (tail vein) with 100 µl of 0.1% Evans blue dye (Anedra, Argentina). The presence of blue color in the skin 30 min following the injection was considered a positive reaction.

#### DTH test

Three weeks after the last boost the delayed-type hypersensitivity (DTH) response was measured by determining footpad swelling after subcutaneous injection of 20 µg of CMP in 20 µl PBS into one hind footpad. As a negative control saline was similarly injected into the contralateral footpad. Footpad swelling was measured 48 h post injection with a digital micrometer with a minimum increment of 0.01 mm.

### In vitro evaluation of allergic disease

#### Serum CMP-specific IgE, IgG1 and IgG2a detection

For the evaluation of specific IgE antibodies against CMP serum samples were tested by EAST as previously described [31]. Serum CMP-specific IgG1 and IgG2a antibodies were measured by ELISA as described [30].

#### Cytokine response of splenocytes to CMP stimulation

Twenty-four hours after the oral challenge mice were killed and spleens were aseptically removed. Splenocytes were cultured at a concentration of 4 x10^6^ cells/well for 72 h at 37 ^°^C in the presence of complete medium or in medium containing CMP (0.35 mg/ml), bovine casein (0.20 mg/ml), or ConA (5 µg/ml) as a positive control. Supernatants were harvested and assayed for IL-5 by ELISA commercial kit (Invitrogen, Invitrogen Corporation, USA).

#### Mucosal gene expression

Jejunum was aseptically removed from mice killed by cervical dislocation 24 h following oral challenge, and mRNA was isolated using illustra RNAspin mini isolation kit according to manufacturer’s specifications (GE Healthcare, Germany). Peyer’s patches were discarded prior to tissue processing. The amount of the extracted RNA was determined by UV absorption and the optical density ratio of OD_280nm_/OD_260nm_ was used as a purity measure. Complementary DNA (cDNA) was obtained by RT-PCR (Invitrogen, Life technologies, USA) and mRNA expression was determined by real-time quantitative PCR. The experimental procedure was performed on *ABI prims* sequence detection system using SYBRGreen fluorescence (BioRad, USA). β-actin was used to standardize the total amount of cDNA, and the fold change in mRNA expression was defined as the ratio of the normalized values corresponding to the sensitized mouse to that of control mouse. Genes of interest were IFN-γ, IL-5, gata-3 and t-bet.

### Statistical analysis

All statistical analyses and plotting were carried out using GraphPad Prism 5 software. T test was conducted if 2 experimental groups were performed, whereas when more than 2 groups were conducted, the significance of the difference was determined using ANOVA test. When data did not fit a Gaussian distribution, a logarithmical transformation was done to achieve a normal distribution. A *P* value <0.05 was considered as statistically significant.

## Supporting Information

Figure S1 Intranasal administration of U-Omp16 does not cause histological changes in lung tissues. Longitudinal sections of the lungs from mice were obtained at 12 h or 2 weeks after i.n administration of (i) PBS or (ii) U-Omp16 (20 µg). Lung histology (10X and 40X right panels) at 12 h after a single administration (**A**) and at 2 weeks (**B**) after administration on days 0 and 7 is shown. Other mice were i.n administered once or in two occasions (day 0 and 7) with (i) OVA (50 µg), (ii) OVA (50 µg) + U-Omp16 (20 µg) or (iii) OVA (50 µg) + CT (1 µg). At 12 or 24 h (C or D) after a single administration and (**E**) 2 weeks after 2 doses, lungs were excised for histological study (*n*/group=5). All images are (10X) and magnifications (40X) are shown in each picture. At the indicated time post administration lungs were excised and fixed in cold sterile *para*-formaldehide 4%. Sections of lungs were obtained and stained with H&E to assess the degree of inflammation or damage in lung structure. Representative pictures from each group are shown.
(TIF)

Figure S2 Intranasal co-administration with U-Omp16 does not re-direct the antigen to CNS. Western blots analysis of OVA and U-Omp16 in different brain areas. (**A**) Protein lysates of olfactory bulb (OB), forebrain (FB) and posterior brain (PB) were obtained from animals i.n administered once with (i) PBS (control), (ii) OVAFITC (50 µg) or (iii) OVAFITC (50 µg) +U-Omp16 (20 µg) at different time points after administration (2, 12 and 24 h). Protein amount in the lysates was quantified by Bradford method and for Western blot experiments 100 µg of total protein were used per lane. (**B**) Western blot analysis of OVA in lysates of OB, FB and PB from mice that were i.n administered with the different formulations (*n*/group=5). (**C**) Suspensions of the different brain areas were obtained and OVAFITC presence was determined in a fluorometer (Victor3, PerkinElmer, Waltham, MA). Data in each row represents the mean of µg OVAFITC/µg of brain area (OB, FB or PB) ±SEM in each analyzed time. Results are representative of two independent experiments. (**D**) Western blot analysis of U-Omp16 in protein lysates of OB, FB and PB from mice that were i.n administered with the different formulations (*n*/group=5). (**E**) **U-Omp16 does not induce inflammation in olfactory bulb**. Animals were i.n. administered once, or on days 0 and 7 with (i) PBS or (ii) U-Omp16 (20 µg) and 12 h or 2 weeks respectively, brains were excised and fixed in cold sterile *para*-formaldehide 4%. Sections of the OB were obtained and stained with H&E to assess the degree of inflammation. Histology of the OB from mice representative from each group (10X) is shown.
(TIF)

Figure S3 Immunization with U-Omp16 induces a Th1 immune response against OVA similar to the one induced by CpG. Mice were immunized by the nasal route with OVA (50 µg) plus i) PBS, ii) U-Omp16 (20 µg) previously digested with proteinase K (U-Omp16PK), iii) U-Omp16 (20 µg) or iv) CpG (10 µg) on days 0, 7 and 14. Three weeks after last immunization animals were sacrificed and spleen cells were stimulated *in vitro* with OVA 500 µg/ml or complete medium. Culture supernatants were harvested 5 days after stimulation and cytokine concentration of (**A**) IFN-γ, (**B**) IL-4 and (**C**) IL-10 (pg/ml) were determined by ELISA. Data represent the mean ±SEM from each group of five mice; (**P<0.01, *P<0.05 *vs* OVA and OVA+U-Omp16PK groups). These results are representative of two independent experiments with similar results.
(TIF)

Figure S4 Nasal administration of U-Omp16 induces recruitment of DCs and monocytes/macrophages in lung tissue. Animals were i.n. administered once with i) OVA, ii) OVA+U-Omp16 or iii) OVA+CT. At different times (2 and 18 h) post administration lungs were excised and cellular suspensions were obtained. Cells (6x10^6^) were stained with specific Abs anti-CD11c, anti-CD11b for flow cytometry analysis (**A**). Data represent the number of cells/lung from administered animals ±SEM (**P<0.01 and *P<0.05 *vs* OVA group). **U-Omp16 induces the production of TNF-α and IL-10 by BMDCs and BMDMs *in vitro***. BMDCs and BMDMs were stimulated for 20 h with different doses of U-Omp16 (20 or 100 µg/ml) or complete medium (control). After *in vitro* stimulation supernatants were harvested and concentrations (pg/ml) of TNF-α and IL-10 were determined (**B**). Data represents means (pg/ml) of duplicate determinations ±SEM (***P<0.001 and **P<0.01 *vs* medium). **U-Omp16 is internalized by DCs *in vitro***. BMDCs (1x10^6^) were incubated for 15 or 30 minutes with U-Omp16FITC labeled (20 or 100 µg/ml) or complete medium (control). After incubation, cells were washed and U-Omp16 fate was determined by flow cytometry (**C**). Data represents the median fluorescence intensity (MFI) ±SEM (***P<0.001 and **P<0.01 *vs* control).
(TIF)
