# Supplementary figures and images for: Unlipidated Outer Membrane Protein Omp16 (U-Omp16) from Brucella spp. as Nasal Adjuvant Induces a Th1 Immune Response and Modulates the Th2 Allergic Response to Cow’s Milk Proteins
Source: PLoS One. 2013 Jul 5;8(7):e69438. doi: 10.1371/journal.pone.0069438 (PMC3703917; doi:10.1371/journal.pone.0069438)

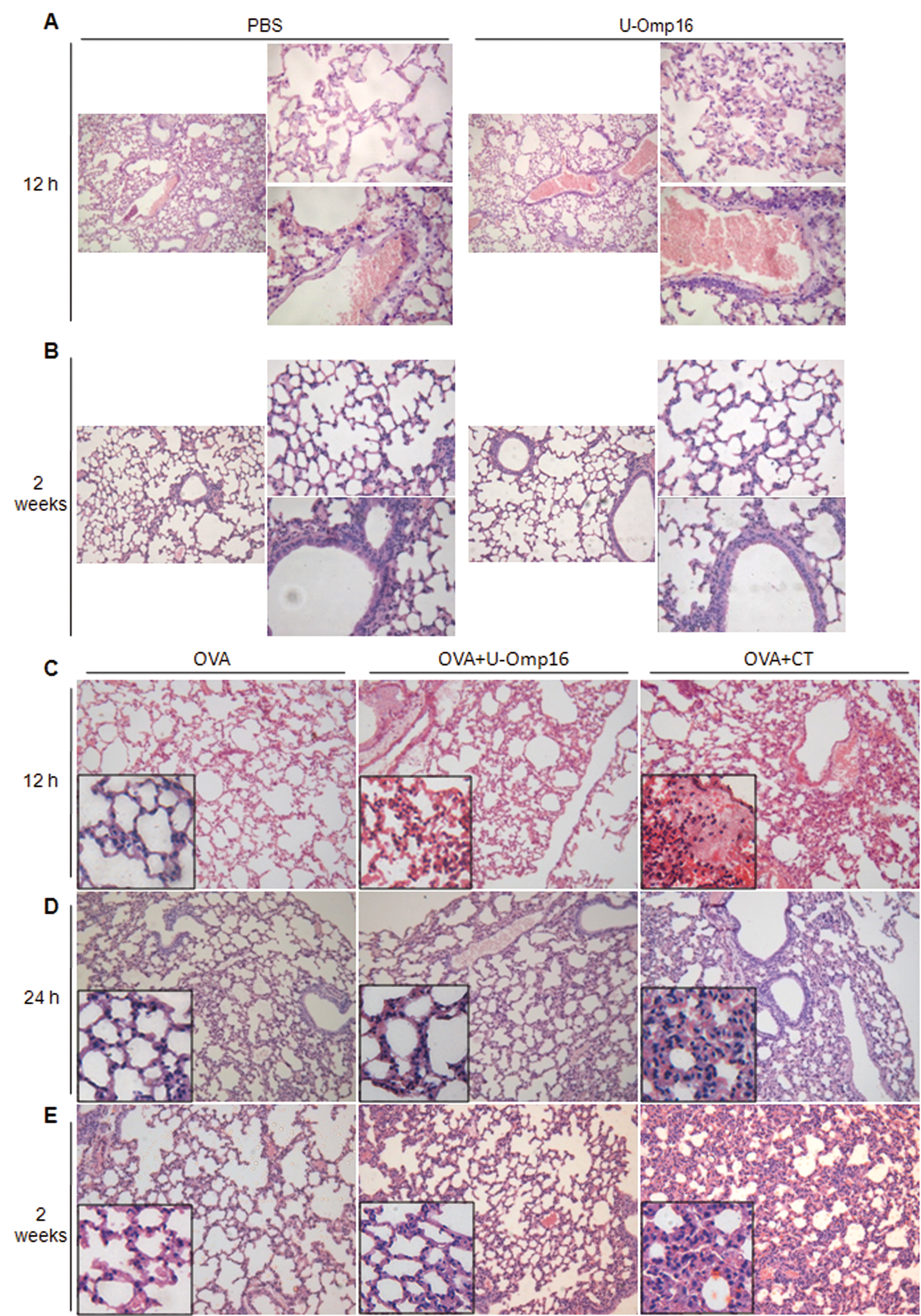

Supplement: Figure S1 — Longitudinal sections of the lungs from mice were obtained at 12 h or 2 weeks after i.n administration of (i) PBS or (ii) U-Omp16 (20 µg). Lung histology (10X and 40X right panels) at 12 h after a single administration (A) and at 2 weeks (B) after administration on days 0 and 7 is shown. Other mice were i.n administered once or in two occasions (day 0 and 7) with (i) OVA (50 µg), (ii) OVA (50 µg) + U-Omp16 (20 µg) or (iii) OVA (50 µg) + CT (1 µg). At 12 or 24 h (C or D) after a single administration and (E) 2 weeks after 2 doses, lungs were excised for histological study (n/group=5). All images are (10X) and magnifications (40X) are shown in each picture. At the indicated time post administration lungs were excised and fixed in cold sterile para-formaldehide 4%. Sections of lungs were obtained and stained with H&E to assess the degree of inflammation or damage in lung structure. Representative pictures from each group are shown. (TIF) [file pone.0069438.s001.tif]

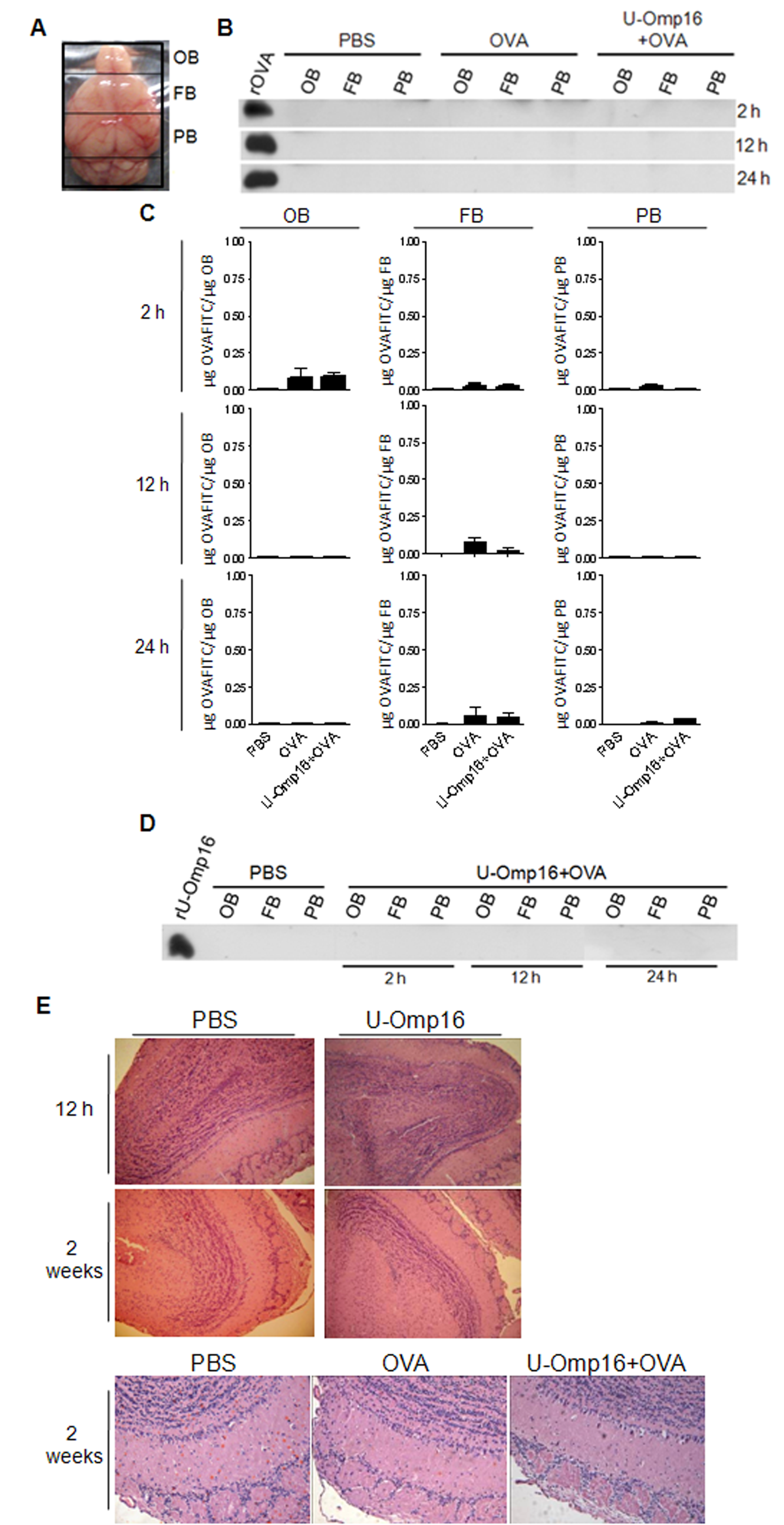

Supplement: Figure S2 — Western blots analysis of OVA and U-Omp16 in different brain areas. (A) Protein lysates of olfactory bulb (OB), forebrain (FB) and posterior brain (PB) were obtained from animals i.n administered once with (i) PBS (control), (ii) OVAFITC (50 µg) or (iii) OVAFITC (50 µg) +U-Omp16 (20 µg) at different time points after administration (2, 12 and 24 h). Protein amount in the lysates was quantified by Bradford method and for Western blot experiments 100 µg of total protein were used per lane. (B) Western blot analysis of OVA in lysates of OB, FB and PB from mice that were i.n administered with the different formulations (n/group=5). (C) Suspensions of the different brain areas were obtained and OVAFITC presence was determined in a fluorometer (Victor3, PerkinElmer, Waltham, MA). Data in each row represents the mean of µg OVAFITC/µg of brain area (OB, FB or PB) ±SEM in each analyzed time. Results are representative of two independent experiments. (D) Western blot analysis of U-Omp16 in protein lysates of OB, FB and PB from mice that were i.n administered with the different formulations (n/group=5). (E) U-Omp16 does not induce inflammation in olfactory bulb. Animals were i.n. administered once, or on days 0 and 7 with (i) PBS or (ii) U-Omp16 (20 µg) and 12 h or 2 weeks respectively, brains were excised and fixed in cold sterile para-formaldehide 4%. Sections of the OB were obtained and stained with H&E to assess the degree of inflammation. Histology of the OB from mice representative from each group (10X) is shown. (TIF) [file pone.0069438.s002.tif]

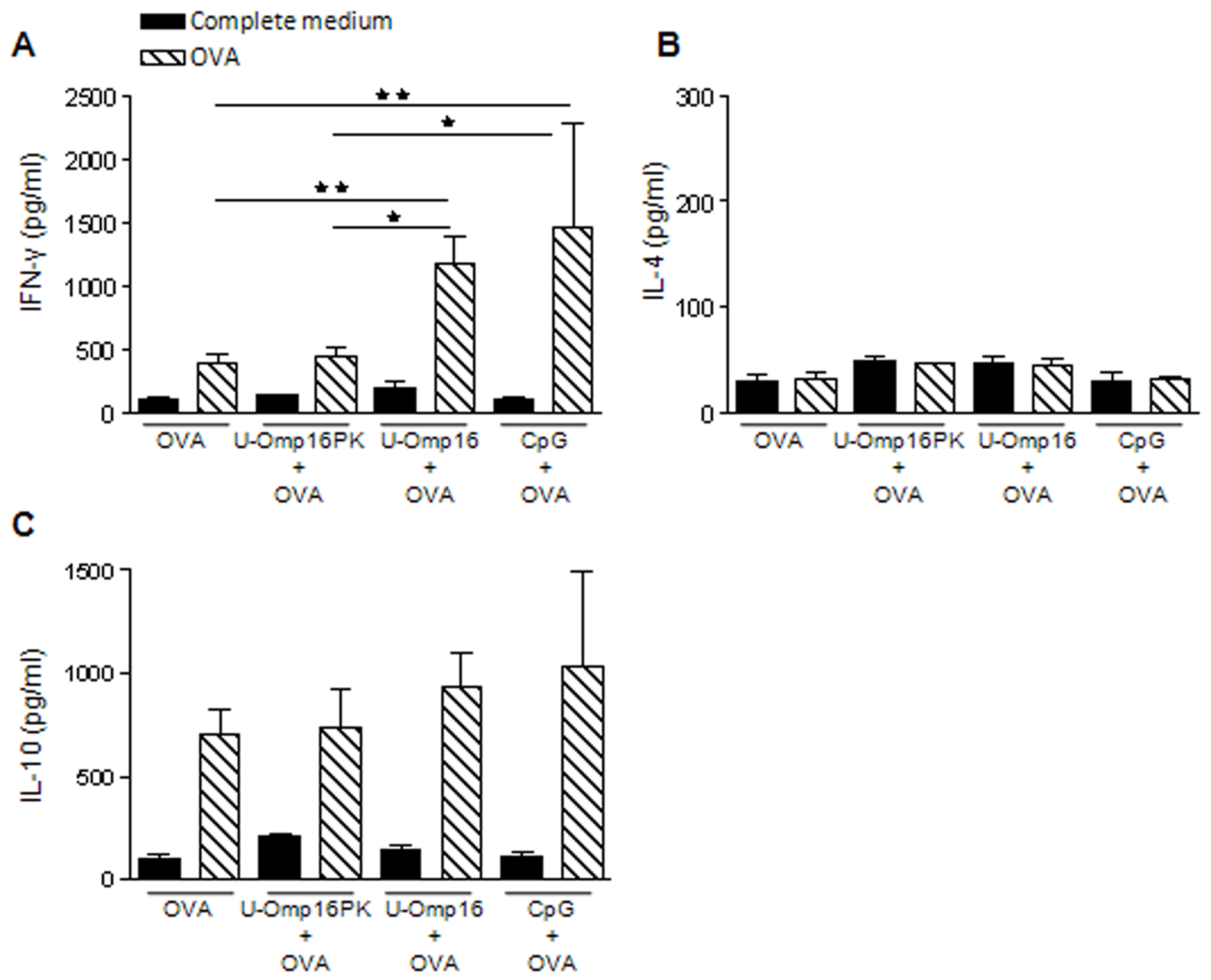

Supplement: Figure S3 — Mice were immunized by the nasal route with OVA (50 µg) plus i) PBS, ii) U-Omp16 (20 µg) previously digested with proteinase K (U-Omp16PK), iii) U-Omp16 (20 µg) or iv) CpG (10 µg) on days 0, 7 and 14. Three weeks after last immunization animals were sacrificed and spleen cells were stimulated in vitro with OVA 500 µg/ml or complete medium. Culture supernatants were harvested 5 days after stimulation and cytokine concentration of (A) IFN-γ, (B) IL-4 and (C) IL-10 (pg/ml) were determined by ELISA. Data represent the mean ±SEM from each group of five mice; (**P<0.01, *P<0.05 vs OVA and OVA+U-Omp16PK groups). These results are representative of two independent experiments with similar results. (TIF) [file pone.0069438.s003.tif]

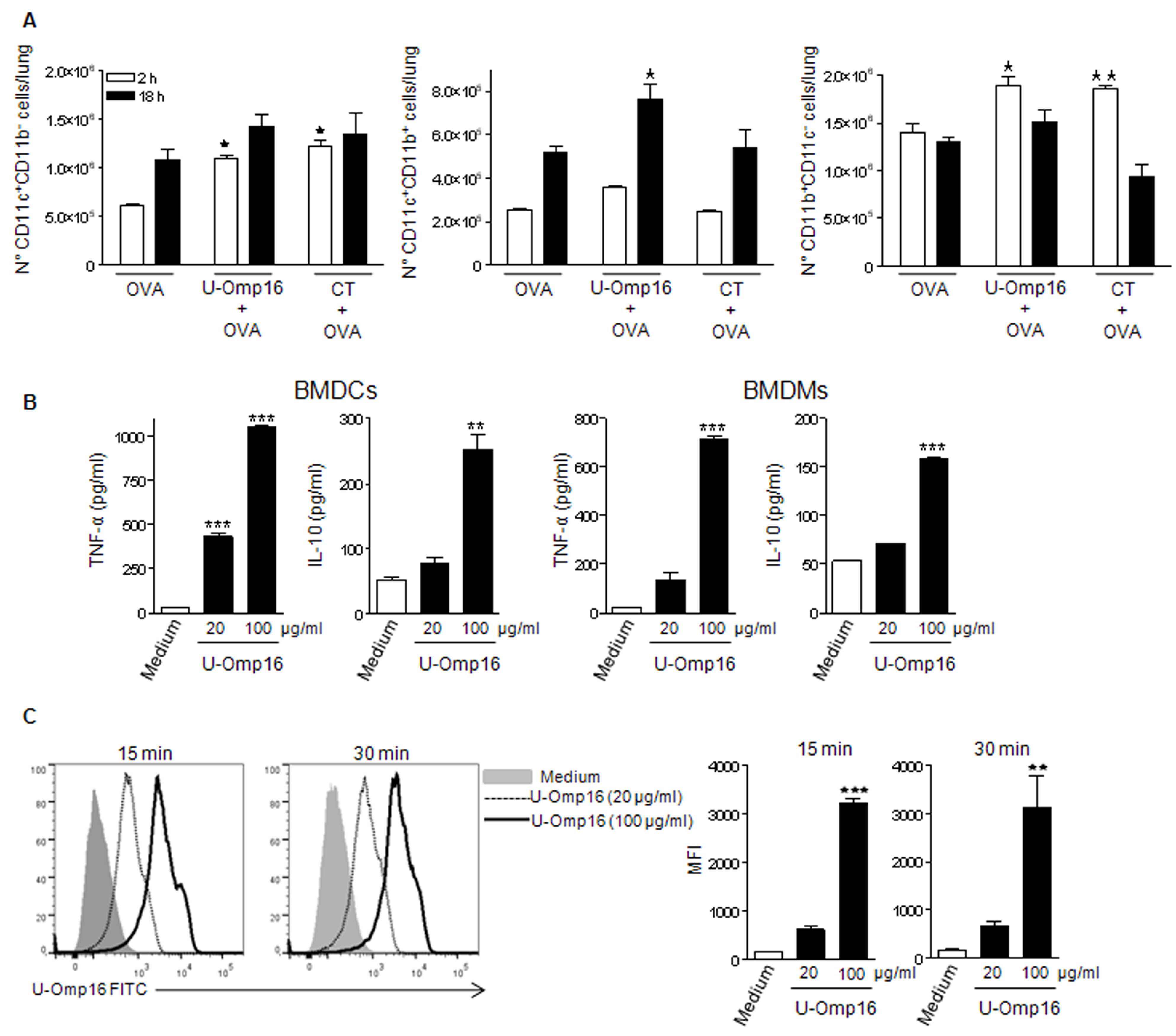

Supplement: Figure S4 — Animals were i.n. administered once with i) OVA, ii) OVA+U-Omp16 or iii) OVA+CT. At different times (2 and 18 h) post administration lungs were excised and cellular suspensions were obtained. Cells (6x10^6^) were stained with specific Abs anti-CD11c, anti-CD11b for flow cytometry analysis (A). Data represent the number of cells/lung from administered animals ±SEM (**P<0.01 and *P<0.05 vs OVA group). U-Omp16 induces the production of TNF-α and IL-10 by BMDCs and BMDMs in vitro. BMDCs and BMDMs were stimulated for 20 h with different doses of U-Omp16 (20 or 100 µg/ml) or complete medium (control). After in vitro stimulation supernatants were harvested and concentrations (pg/ml) of TNF-α and IL-10 were determined (B). Data represents means (pg/ml) of duplicate determinations ±SEM (***P<0.001 and **P<0.01 vs medium). U-Omp16 is internalized by DCs in vitro. BMDCs (1x10^6^) were incubated for 15 or 30 minutes with U-Omp16FITC labeled (20 or 100 µg/ml) or complete medium (control). After incubation, cells were washed and U-Omp16 fate was determined by flow cytometry (C). Data represents the median fluorescence intensity (MFI) ±SEM (***P<0.001 and **P<0.01 vs control). (TIF) [file pone.0069438.s004.tif]
